# Nanodelivery of Dietary Polyphenols for Therapeutic Applications

**DOI:** 10.3390/molecules27248706

**Published:** 2022-12-08

**Authors:** Mithun Rudrapal, Ashwini K. Mishra, Laxmi Rani, Khomendra K. Sarwa, James H. Zothantluanga, Johra Khan, Mehnaz Kamal, Santwana Palai, Atul R. Bendale, Swati G. Talele, Vasim T. Pathan, Laxmikant B. Borse, Vishnu S. Neharkar, Pravat K. Sahoo

**Affiliations:** 1 Department of Pharmaceutical Chemistry, Rasiklal M. Dhariwal Institute of Pharmaceutical Education and Research, Pune 411019, India; 2 Department of Pharmaceutics, Delhi Institute of Pharmaceutical Sciences and Research, Delhi Pharmaceutical Sciences and Research University, New Delhi 110017, India; 3 Department of Pharmacy, School of Medical and Allied Sciences, GD Goenka University, Gurugram 122103, India; 4 Department of Pharmacy, Government Girls Polytechnic, Raipur 492001, India; 5 Department of Pharmaceutical Sciences, Faculty of Science and Engineering, Dibrugarh University, Dibrugarh 786004, India; 6 Department of Medical Laboratory Sciences, College of Applied Medical Sciences, Majmaah University, Al-Majmaah 11952, Saudi Arabia; 7 Department of Pharmaceutical Chemistry, College of Pharmacy, Prince Sattam Bin Abdulaziz University, Al-Kharj 11942, Saudi Arabia; 8 Department of Veterinary Pharmacology and Toxicology, College of Veterinary Science and Animal Husbandry, Orissa University of Agriculture and Technology (OUAT), Bhubaneswar 751003, India; 9 Sandip Institute of Pharmaceutical Sciences, Nashik 422213, India; 10 Department of Pharmacology, Rasiklal M. Dhariwal Institute of Pharmaceutical Education and Research, Pune 411019, India

**Keywords:** dietary polyphenols, nanotechnology, nanoformulation, nanophytomedicine, nanodelivery

## Abstract

Advancement in nanotechnology has unleashed the therapeutic potentials of dietary polyphenols by enhancing bioavailability, improving biological half-life, and allowing site-specific drug delivery. In this review, through citation of relevant literature reports, we discuss the application of nano-pharmaceutical formulations, such as solid lipid nanoparticles, nano-emulsions, nano-crystals, nano-polymersomes, liposomes, ethosomes, phytosomes, and invasomes for dietary polyphenols. Following this, we highlight important studies concerning different combinations of nano formulations with dietary polyphenols (also known as nanophytopolyphenols). We also provide nano-formulation paradigms for enhancing the physicochemical properties of dietary polyphenols. Finally, we highlight the latest patents that were granted on nano-formulations of dietary polyphenols. Based on our review, we observe that nanosized delivery of herbal constituents, spices, and dietary supplements have the ability to improve biological processes and address issues connected with herbal treatments.

## 1. Introduction

Nano-medicine and nano-drug delivery systems have been a continuously emerging aspect of science, wherein constituents of nano-scale range are actively utilized as diagnostic kits and/or to deliver active pharmaceutical agents at the site of action, with the release controlled as desired. Since ancient times, herbal remedies or dietary components have been extensively utilized all over the world and acknowledged by physicians and patients for their superior therapeutic impact over allopathic medicines. The reason for such usage is the minimal side effects compared to chemical therapeutic agents used in modern medicinal systems [1,2]. Drug delivery methods can accomplish herbal or spice treatments. This herbal remedy contributes to the therapeutic benefit of medications by lowering their toxicity and unwanted effects, as well as boosting their gastrointestinal absorption and metabolic effects [3]. Nanotechnology plays a crucial part in the current strategy, and its usage in herbal medicinal systems, specifically drug delivery systems, is expected to grow fast. Herbal drugs utilized in novel delivery systems have the ability to improve the pharmacological profile and overcome the issues connected with medicinal or herbal plants in the future [4]. Therefore, herbal nano-carriers will help to confront deadly diseases like cancer, hypertension, diabetes, and other diseases. Recent research and developments in the arena of nanotechnology have revealed the delivery of herbal therapeutic molecules to their specific site in order to treat various critical diseases [5,6]. In recent years, along with the application of nanotechnology in health and the environment, numerous herbal medicine-based delivery methods have been effectively used. Nanopolymers have many applications in health and the environment. It causes serious health concerns as well. For example, nanoplastics can cause adverse effects or toxicities on human health and the environment [7]. The physicochemical properties of nanoformulations (nano-emulsion, nano-encapsulation, polymeric nanoparticles (NPs), nano-liposomes, solid liquid nanoparticles, etc.), such as shape, size, and surface properties, are responsible for their improved biological properties, including toxicities [8]. Though dietary polyphenols have good protective function, they lack efficient delivery into the body due to their poor bioavailability and metabolic instability. Nanoformulation of polyphenols can overcome such problems and can be efficiently delivered for the treatment of cancer, as well as neurological and cardiovascular diseases [9,10]. Bio-based nanocarriers can be designed for encapsulating, protecting, and delivering polyphenols, thereby improving their oral bioavailability, which find wide application as formulations in health and nutrition products, including nuraceuticals [11]. Although some problems remain, improved technology must be designed to ensure the effective delivery of medications to their specified target areas. As a result, nano-drug delivery systems are now being researched in order to assist enhanced herbal drug- or phytocomponent-based delivery systems.

## 2. Nanodelivery Systems for Herbal/Phytoconstituents

Herbs and spices have been historically characterized as plant components utilized in the diet for their fragrant characteristics, but have no or minimal nutritional value. Herbs and spices, on the other hand, have been found as sources of different phytochemicals with strong antioxidant activity. As a result, phytoconstituents from herbs and spices can be formulated in various forms of nano-drug delivery systems. The nanodelivery systems for various phytoconstituents are discussed below.

### 2.1. Solid-Lipid Nanoparticles

Solid-lipid nanoparticles (SLNs) are concurrent to nano-emulsions with the minor difference of having lipids playing the role as a solid phase. SLNs are the sub-micron (50–1000 nm)-sized colloidal nano-carriers composed of physiologically compatible lipids dispersed in an aqueous solution having surfactants dissolved in it. Some of the methods by which SLNs are prepared are micro fluidization (also called high-pressure homogenization) and ultra-sonication [12]. The merits of SLNs are that they are of submicron size, providing a large surface area, increased drug payload, and enhanced interfacial interaction. Colloidal nanocarriers such as nanoemulsion, microemulsions, polymeric nanoparticles, and liposomes do not provide efficient targeting effects on the site of action and controlled release profile, and these are some of the limitations encountered by SLNs. Drug payload is achieved in two ways. Firstly, the drug can be integrated into the polymeric core, and secondly, it can be attached to the surface of the polymeric part. A major advantage perceived in SLNs is that they entrap the lipophilic drug in a stable form without the use of harmful organic solvents [13]. Other advantages of SLNs include their unique size-related aspects and their ability for drug incorporation; both lipophilic and hydrophilic drugs can be loaded. Large scale production is easier to achieve, as well as higher bioavailability with minimal toxic effects.

### 2.2. Nano-Emulsions

Nano-emulsions include mixtures of two or more immiscible liquids, of which one is water and the other may be any type of oil used in the formulation. These types of nano-formulations are prepared from chemical or mechanical process. When a chemical process is used, nanoemulsion droplets are formed as a result of the interface of hydrophobic compounds with emulsifiers, whereas a mechanical process includes high shear homogenization of large emulsion droplets to form nano-droplets. A basic concept which differs from conventional emulsion with nanoemulsion is the size and shape of emulsion droplets formed (ranging from 20–200 nm) [14].

### 2.3. Nano-Crystals

Nano-crystals are sub-micron (20 to 850 nm)-sized colloids dispersed in a dissimilar phase consisting of drug molecules. These nano-crystals are also formulated using chemical or mechanical methods, with an advantage of reduced particle size within a nano-range and provide increased surface area to remain in contact with the dissolving phase. Some of the merits of nanocrystals include improved saturation solubility and dissolution rate with greater drug loading, which provide more benefits compared to conventional dosage forms [15].

### 2.4. Nano-Polymersomes

Nano-polymersomes (NPS) are vesicles of polymers with a size ranging between 10 nm to 1 µm, which are forms of an aqueous core as a result of self-assembling amphiphilic copolymers. The tuneable properties of NPS allow for flexible biomedical advantages; e.g., as drug delivery vehicles or as artificial organelles. The synthetic process is the same as polymeric nanoparticles [16]. In case of NPS, a wide variety of biodegradable and stimuli-responsive polymers are utilized in drug encapsulation and improving the release behavior owing to their tuneable properties. NPS have an excellent capability in incorporating both types of drug molecules; i.e., lipophilic (in the polymeric membrane bilayer) and hydrophilic (in the aqueous core). The major advantages over nano-lipid carriers (NLCs) are that NPS provide a more stable vesicular formulation, a wide range of usages, and a controlled drug release profile [4,5].

### 2.5. Liposomes

**Figure 1:**
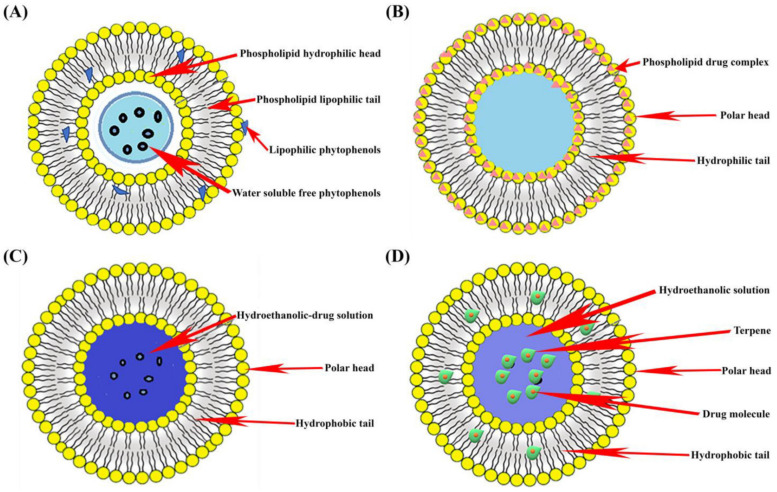
Graphical representation of different types of nanodelivery systems: (**A**) Liposome; (**B**) Phytosome; (**C**) Ethosome; and (**D**) Ivasome.

Liposomes are biodegradable sphere-shaped phospholipid bilayer vesicles with an aqueous core (Figure 1). They are formed by the self-assembly of phospholipids. The organized structure of liposomes allows the loading of hydrophilic drug molecules in the inner aqueous core and lipophilic drug molecules into the phospholipid bilayer. Liposomes are synthesized by different conventional methods, some of which are the thin film hydration method, reverse phase evaporation, the solvent injection method, and the detergent removal method. Liposomes are categorized as unilamellar, multilamellar, and multivesicular vesicles based on the size of the vesicle and number of phospholipid bilayers [17,18,19]. From the literature search, it was found that liposomal forms of polyphenols have improved therapeutic benefits. Researchers reported that catechin liposomes showed increased bioavailability and cerebral distribution when compared to catechin alone [20]. Curcumin liposomes have a prolonged antioxidant effect compared to the uncomplexed curcumin [21]. Researchers have developed a quercetin liposomal formulation and observed that the formulation showed increased solubility, bioavailability, and in vivo antitumor efficacy [22,23,24].

### 2.6. Ethosomes

Ethosomes are phospholipid-based vesicular carriers composed of a high concentration of ethanol (Figure 1). These vesicles were first developed by Touitou and co-workers in 1997 for the efficient delivery of active agents across the skin. Ethosomes are a non-invasive carrier system. The higher amount of ethanol in ethosomes provides a negative charge on the skin surface and increases its permeability, which in turn facilitates therapeutic agents to reach the deeper skin tissues for systemic circulation. Ethosomes are developed by different methods, such as the mechanical dispersion method, the cold method, the hot method, etc. [25,26].

The incorporation of phytopolyphenols into ethosomes has been considered a deliberate approach to increasing in situ stability, the permeability of polyphenols through the skin, bioavailability, and therapeutic efficacy. Researchers have encapsulated epigallocatechin3-gallate (EGCG) in ethosomes and observed an improved antioxidant activity and photostability of EGCG [27,28]. Researchers prepared apigenin-loaded ethosomes and found that ethosomal formulation showed higher in vivo skin targeting and effectiveness against ultraviolet B radiation-induced skin inflammation in comparison with its liposomal formulation [29,30].

### 2.7. Phytosomes

Phytosomes are phospholipid and herbal drug extract complex vesicles in which phytoconstituents are bound by a phospholipid layer (Figure 1). Phospholipids have the property of an active emulsifier because of their hydrophilic head and lipophilic tail. The emulsifier property of phytosomes improves phytopolyphenol’s bioavailability by facilitating them to pass from the aqueous to the lipophilic environment of the cell membrane [31]. Researchers have reported improved hepatoprotective and antioxidant effects of silybin phytosomes formulation in comparison to silybin alone [32]. Researchers have reported ginkgo phytosomes and their improved effect on the brain and vascular protection. Researchers have also prepared rutin phytosomes transdermal formulation and found its remarkable effect in rheumatoid arthritis [33,34].

### 2.8. Invasomes

Invasomes are nano-vesicular transdermal drug delivery systems, an ethanol and terpenes mixture with phospholipids (Figure 1). By breaking down lipid packing in the stratum corneum, invasomes enhance drug permeability and retention into the skin layers for localized effect [35]. Invasomes are mostly prepared by mechanical dispersion technique and film hydration technique. Invasomes, as a drug carrier for phytopolyphenols, have the advantage of enhanced solubility and therapeutic effect [36]. Researchers have developed an invasomal cream of *Ocimum basilicum* for the treatment of acne and observed successful drug delivery through the skin [37].

## 3. Nanodelivery of Dietary Polyphenols

### 3.1. Nano-Resveratrol

**Figure 2:**
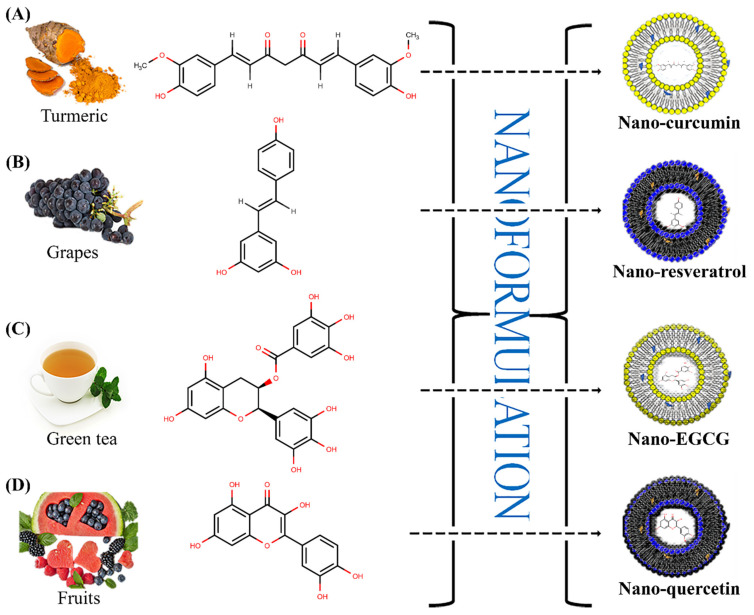
Graphical examples highlighting the concept of nano-phytopolyphenols with selected dietary polyphenols and their common natural sources; namely (**A**) nano-curcumin, (**B**) nano-resveratrol, (**C**) nano-EGCG, and (**D**) nano-quercetin.

Resveratrol (3,5,4′-*trans*-trihydroxystilbene) exists as a polyphenolic compound formulated by grape skin and seeds, and, in lesser amounts, in several additional sources such as apples, plums, and peanuts. It serves as a phytoalexin in herbal plants, defending them from microbial and fungal attacks. The potent antioxidant properties of resveratrol in many animal models have been constantly conveyed [38]. The antioxidant activity of resveratrol is greatly influenced by the redox characteristics of phenolic hydroxyl groups, as well as the possibility for electron delocalization throughout their chemical structure. Resveratrol has three -OH groups (as described in Figure 2) that are well known for reactive oxygen species (ROS) scavenging potential. Furthermore, these -OH groups aid in the chelation of metals or ions, which inhibits ROS generation. Further, resveratrol does not only work as a direct antioxidant system, but also indirectly acts as an endogenous antioxidant system inducer, which may contribute to cellular defense. Additionally, these polyphenolic components’ antioxidant properties may be due to their functions as gene regulators [1,8]. It was discovered that the inhibition of ROS generation was achieved by down regulating the expression of NADPH oxidase. When mitochondrial biogenesis was stimulated by resveratrol, a reduction in mitochondrial superoxide was observed as a function of tetrahydrobiopterin-synthesizing enzyme GTP cyclo-hydrolase-I, which results in increased antioxidant enzymatic levels. Most of the health benefits are observed because of the antioxidant effects possessed by resveratrol. Recently, some nano-sized resveratrol-loaded delivery systems have been highlighted by researchers due to their potential clinical usage. One investigation showed that resveratrol-loaded nano-capsules showed 2-fold higher bioavailability compared to free drug in visceral organs (brain, liver, and kidneys) of male Wistar rats [39]. Nano-formulations of resveratrol exhibited safer and improved absorption of the drug in this model. The folate-conjugated human serum albumin-encapsulated resveratrol polymeric nanoparticles demonstrated the bioavailability of the drug is increased by 6-folds compared to intravenous administration of the raw form of resveratrol [40]. Resveratrol liposomes have improved solubility and chemical stability compared to resveratrol alone. Researchers prepared hybrid liposomes and found that the formulation showed increased bioavailability, stability, and enhanced liver protection. These preliminary findings suggest that liposomes might be used as a successful delivery system for phytopolyphenols in pharmaceutical applications [41].

Authors have reported that resveratrol solid lipid nanoparticles could cross the cell membrane in keratocytes, as the resveratrol is strongly bonded with lipid molecules which help enhance the penetration of nano-resveratrol along with the sustained drug release effect [42]. Another study was performed in a xenograft ovarian cancer diseased mice model, in which nano resveratrol bound with serum albumin showed a better solubility profile, which finally resulted in increased inhibition of tumor cell growth [43]. Shao et al. [44] reported methoxy-PEG-PCL-based nano resveratrol formulation showed higher cytotoxic effects against tumor cells compared to free drug due to increased cellular uptake of nano resveratrol.

### 3.2. Nano-Curcumin

Curcumin obtained from rhizomes of turmeric (*Curcuma longa*) has been traditionally used as a spice and dietary supplement in Asian countries, and as a polyphenolic compound also possesses medicinal properties. Importantly, it is composed of three structural components in an aryl hydrocarbon skeleton, of which one is a diketone and the other two are phenolic groups, as demonstrated in Figure 2.

The oxidation of these functional groups transfers electrons and undergoes hydrogen abstraction to produce an antioxidant effect. Methylene hydrogen and o-methoxy phenolic groups are responsible for such an effect. Additionally, the β -diketone groups may chelate transition metal ions, and such complexes formed mimic enzymatic antioxidant activity. Furthermore, curcumin also has strong anti-inflammatory and anticancer activities, as well as neurodegenerative action [21]. Evidential reports have shown that curcumin is used in age-related disorders, inflammation, atherosclerosis, oxidative stress, cardiovascular diseases, type-2 diabetes, rheumatoid arthritis, and ocular diseases. When it comes to PLGA-based nano-formulations of curcumin, reports have shown 22-fold improved oral bioavailability than that of the raw form of curcumin. Curcumin-loaded SLNs showed 16 times higher bioavailability in cerebral an ischemic rat model. When the bioavailability of N-trimethyl chitosan surface-modified SLNs were compared with that of the raw form of curcumin, it was found that SLNs showed enhanced drug distribution in the brain and improved oral bioavailability [45]. Nano-capsules of curcumin prepared by Eudragit RL100 polymer were reported to have greater antioxidant potential with low lipid peroxidation in the milk of dairy sheep [46]. Researchers reported enhanced anti-inflammatory and antioxidant effects of curcumin invasomes in comparison to other vesicular systems. Nanocomposites loaded with curcumin showed greater anti-inflammatory property, as mentioned in the reported literature [47].

Exir Nano-Sina Co. (Iran) [48] developed a nano-curcumin system that could improve the stability and bioavailability of curcumin, along with improved water solubility, safety, biodistribution, and bio-compatibility. Such nanodelivery could reduce the dosage of curcumin to one-fifth of the original dose to achieve the desired anti-inflammatory effects in clinical trials. Studies have also reported that the highest anti-inflammatory efficacy (from clinical results) was observed by curcumin among all polyphenols when curcumin was administered in a modified dosage form in the nanodelivery system. In a meta-analysis by Damoon et al. [49], nano-curcumin could significantly increase HDL levels, decrease inflammatory markers, and improve the glycemic profile with cardioprotective effects. In open, non-randomized clinical trials, the efficacy of nano-curcumin oral formulation was assessed on patients with moderate COVID-19. This clinical investigation revealed that curcuminoids as nano micelles significantly improved recovery time from symptoms, such as myalgia, fever, and tachypnea, with a treatment duration of 14 days [50]. Nano-curcumin in combination with omega-3 fatty acids mutually strengthened activity in order to down regulate the COX/iNOS mRNA gene in neuroinflammation which could offer symptomatic relief to migraine patients [51].

### 3.3. Nano-Genistein

Genistein [4′,5,7-trihydroxyisoflavone or 5,7-dihydroxy-3-(4-hydroxyphenyl) chromen-4-one] has been proven to be a potent antioxidant flavonoid. The two enzymes responsible for antioxidant properties, namely sodium oxide dismutase (SOD) and catalase (CAT), were induced through gene expression by genistein. In the treatment of some major disorders, such as osteoporosis, obesity, cancer, neurodegeneration, and type-2 diabetes, this isoflavone has been utilized. Some clinical limitations have also been encountered because of its low bioavailability [52]. Some adverse effects, such as endocrine disruption and toxic effects, were also reported when used in higher dose. Nanotechnology has been proven to be useful in encountering such limitations, for example, genistein-loaded polymeric nano micelles have previously showed a better plasma profile and bioavailability compared to free genistein. Some previous investigations have determined that the higher solubility and GI release of drugs from nano-micellar genistein formulations are the reason for enhanced oral bioavailability. Similar results were observed when the bioavailability profile of genistein-loaded SLNs were compared with bulk drug formulations (e.g., suspensions and powders) [53]. Comprehensive data, which includes both clinical and pre-clinical studies of nano-genistein, noted increased tumor cell growth inhibition with different signaling pathways and differentiations of colorectal cancer [54]. Dev et al. [55] showed that genistein nanoparticles (GLNPs) effectively suppressed poly-comb protein expression and slowed the development of oral squamous cell carcinoma by using lactalbumin as a carrier.

### 3.4. Nano-EGCG

In green tea (*Camellia sinensis*), one of the major phyto-phenol is (-)-epigallocatechin-3-gallate (EGCG) (see Figure 2 for illustration). EGCG reported various pharmacological health benefits, such as antioxidants, tumor chemoprevention, refining heart health, enhancing weight loss, shielding the skin from the impairment caused by ionizing fallout, and many more. Many nanoformulations have come under consideration to improve transport capacity, solubility, bioavailability, tumor targeting efficacy, and stability in the gastric environment and systemic circulation. Such an approach reduces the risk of adverse events seen in patients. Inflammation is known to be a major challenge, as it is responsible for the occurrence of several diseases [48]. Nanoscale poly (lactide-co-glycolic acid) (PLGA) particles incorporating EGCG were prepared and their response toward inflammation in human dermal fibroblasts was assessed, which showed a persistent inhibitory response compared to crude EGCG. EGCG phytosomes also showed a significant anti-inflammatory response against acute paw edema in a rat model prepared using an experiment with higher drug loading and better physical stability. EGCG polyphenol has antioxidant and anti-aging effects against UV radiation. A potential pharmacological response was seen by hyaluronic acid combined with nano-transferosomes through the thin film hydration technique [56]. The optimized nano-transferosomes increases cell viability and reduces ROS, as well as matrix metalloproteinases in keratinocyte cell lines. EGCG has cancer chemoprotective potential and anti-proliferative effects, which significantly arrest the G1 phase of the cell cycle and apoptosis. Improved antitumor activity and reduced side effects were seen in stable EGCG-nanoemulsion on lung cancer, which was determined by MTT assay. Another study performed by researchers in Ehrlich tumor-bearing mice by efficiently delivering EGCG gold nanoparticles to tumor sites prepared by simple green synthesis obtained results that were significant compared to the pristine EGCG. Other nanodelivery carriers have also been highlighted, which show great potential in improved delivery of EGCG, such as polymeric nanoparticles, liposomes, and SLN [57,58]. Cell culture, animal experiments, epidemiological studies, and clinical trials showed that EGCG inhibited tumor growth [59,60]. The ability to suppress vascularity, tumor development, and proliferation was substantiated by in vivo research in mice model [61].

### 3.5. Nano-Kaempferol

Kaempferol is found in various types of commonly found fruits and vegetables; for example, cabbage, broccoli, grapes, strawberries, sprouts, and apples. The anti-inflammatory response exhibited by kaempferol is being utilized in the treatment of various diseased conditions, such as intervertebral disc degeneration, colitis, post-menopausal bone osteoporosis, acute lung damage, different types of cancer (breast cancer, hepatocellular carcinoma, esophageal cancer, ovarian cancer, cervical cancer, gastric cancer), diabetes, and fibroproliferative disorders. Bioavailability, solubility, adverse drug events, and site-specific targeting for kaempferolhas must be achieved to improve patient compliance and efficacy. Poly (ethylene oxide)-poly (propylene oxide)-poly (ethylene oxide) (PEO-PPO-PEO), and poly (DL-lactic acid-co-glycolic acid) (PLGA) nanoparticles showed a significant decrease in tumor cell viability, as better bioavailability was achieved compared to crude kaempferol [27,28]. Nanostructured lipid carriers (NLC) loaded with kaempferolwere tested against MDA-MB 468 breast cancer cells, and the cytotoxic effects were studied with MTT assay. Kaempferol NLCs showed an amplified effect with paclitaxel on breast cancer cells by obstructing apoptotic signaling and clampdown of tumor cell cycle arrest. Kaempferol gold nanoclusters with an optimization of morphological characteristics and its anticancer properties were explored in A549 lung cancer cells, which revealed that efficient conjugation of kaempferol with gold nanoclusters was found toxic to cancerous cells, but not lethal towards HK-2 normal human cells. Kaempferol nanoparticles showed cardiovascular protective effects against fluorouracil. The nanoparticles possessed a significant reduction in elevated cardiac enzymes, vascular endothelial growth factor expression, oxidative stress, and cardiac tissue increase, which were confirmed by histopathological studies [62]. A layer-by-layer nano-matrix of kaempferol was prepared and optimized to attain efficient bone formation by retaining the drug in bone marrow for a longer time. This increased bioavailability, boosted the anabolic effect in osteopenic rats, and increased the plasma and bone marrow concentration of kaempferol compared to the crude form of kaempferol. These nanocarriers can improve medication bioavailability, efficiently gather in the tumor location, promote tumor cell uptake, integrate therapeutic agents with imaging tools, and enhance antitumor properties [62,63]. Nano-kaempferol is a budding agent in the management of various diseases. Clinical studies noted anti-inflammatory, type 2 diabetes, and cardiotonic effects for nano-kaempferol, suggesting that it alleviated the levels of markers, such as IL-6, TNF-α, and IL-8 [64].

### 3.6. Nano-Naringenin

Naringenin (NR) is found in citrus fruits such as bergamot, tomatoes, and cherries. NR has a variety of pharmacological activities, including anti-tumor, anti-inflammatory, and anti-oxidant. However, therapeutic efficacy is hampered due to its high hydrophobic property, which can be overcome by incorporating naringenin in nanodelivery carriers such as micelles, liposomes, SLN, nanosuspensions, and many more, as previously discussed. It has been reported that amelioration of anti-inflammatory activity in Freund’s adjuvant arthritis model was achieved by sustained-release NR nanoparticles with enhanced oral bioavailability, GIT absorption, and solubility profiles. The adopted polymeric system controls the rate of release, which was up to 80% release in 24 h, and exhibits prolonged circulation which ultimately diminishes arthritic inflammation. Eudragit E100 Cationic polymeric nanoparticles of NR showed improved absorption, increased bioavailability up to 96 folds, and increased anticancer potential up to 16 folds in in vitro and in vivo investigations. A mechanistic approach of NR conjugated with a PLGA doxorubicin nanoparticulate system has shown well-improved efficacy, a coactive enhanced effect, and reduced toxicity [65,66]. An in vitro study showed better selective anti-tumor potential for breast cancer. An in vivo tumor cell toxicity assay of the NR nanoformulation stopped the tumor growth in an animal model. NR was proven to be effective in the management of parkinsonism disease. Intranasal delivery of NR-Vitamin Eloaded nanoemulsion was administered in rats. The results revealed the behavioral activity of rats reverted to normal, which was supported by higher glutathione levels with a significant lowering of malonaldehyde in the brain tissue. The NR-loaded sulfobutylether-β-cyclodextrin/chitosan nanoparticles were demonstrated as a potential substitute for ocular delivery of poorly soluble NR and gave a sustained rate of release with no irritating activity towards the rabbit’s eye [67]. Oxidative stress induced due to cadmium in Nile tilapia fish was attenuated by NR nanoparticles of an average size range of 165.1 nm due to enhanced antioxidant potential. Cadmium induces oxidative stress in fish, which significantly increases hepatic malondialdehyde and other antioxidant enzyme levels in kidneys and hepatocytes. There was a significant lowering of such enzymes due to the bioaccumulation of nano-NR in the liver and kidney cells. When synthesized in the right nanostructures, NR is a possible therapeutic alternative for the treatment of numerous diseases, including cancer, neurodegenerative problems, liver diseases, ophthalmic disorders, inflammatory diseases, skin diseases, and diabetes [68]. A randomized, placebo-controlled clinical investigation of nano-naringenin imparted hepatoprotective effects in obese patients. Some of the secondary effects could lower blood pressure and improve metabolism [69].

### 3.7. Nano-Apigenin

Flavonoids are the largest group of polyphenols found in plants, in which apigenin (AG) is a remarkable bioactive compound. Many researchers have used apigenin in different conventional and nanodelivery systems. The apigenin has ameliorating effects on various diseases like diabetes, Alzheimer’s disease, dementia, cancer, insomnia, inflammatory condition, and many more. Anti-diabetic activity against Type 2 diabetes was higher when treated by AG-bilosomes compared to simple AG dispersion, which was confirmed by an improved biochemical parameter. An optimized AG bilosomes formulation, with a vesicle size of 183.25 nm, provided enhanced release and permeation with 4.49 times higher flux. A tumor cell targeted by AG nano assemblies with high drug loading and entrapment efficiency showed high expression of CD44 receptors. These AG nanoassemblies facilitate enhanced internalization of AG in tumor cells as affinity on CD44 receptors was greater, and also the formulation provided sustained release along with a longer retention time in the blood [70,71]. The PLGA-loaded AG nanoparticles were designed and formulated, and their efficacy toward ultraviolet-induced skin cancer was assessed. Nano-AG showed promising anti-cancer potential by amelioration of mitochondrial matrix swelling due to enhanced penetration of carriers in tissues. AG nanoparticles showed protective effects against hepatocellular carcinoma in rat models. This class of nano-AG bears a sustained release profile with an enhanced affinity towards the liver cancer cells. Pharmacokinetics and biodistribution studies have also revealed a significant increase of AG in systemic circulation, which proves the futuristic potential for liver cancer patients. Nano-AG bears an impactful pharmacological activity towards various types of cancer, such as breast cancer, skin melanoma, and lung cancer. Rheumatoid arthritis is an immunological disorder in which chronic synovitis leads to inflamed joints and tissue dysfunction. This was managed with SLN highly loaded (80.44% drug encapsulation) with AG, with an optimized particle size of almost 161 nm. The study showed a greater amount of AG release; enhanced permeation and stable mucoadhesiveness by chitosan coat leads to improved bioavailability. The antioxidant potential also remarkably increased in AG-loaded mucoadhesive SLN [72]. Many clinical trials on the nano-apigenin showed remarkable activity against different types of cancers [73]. Nano designing could also be used to enhance the solubility and bioavailability profile of apigenin, which showed promising results against breast cancer cell growth [74].

### 3.8. Nano-Theaflavins and -Thearubigins

Theaflavins (TF) and thearubigins (TR) are natural polyphenolic compounds obtained from catechins through enzymatic oxidation of tea (*Camellia sinensis*). Today, these compounds are utilized in the green synthesis of nanoformulations. Gold-nanoparticles were synthesized with tea leaves involving the chemistry of green synthesis nanotechnology concepts, which can be utilized for antibacterial activity and enhanced antioxidant properties. Silver nanoparticles were also synthesized with green chemistry principles with TF and TE to improve their bactericidal action against *Salmonella typhi* with antibiotics. TE and TF act as stabilizing agents for silver nanoparticles. Reduced absorption and bioavailability of TE and TF are related to a bulky stearic structure, low permeability, poor stability, and active effluxes from the gastrointestinal tract (GIT). To overcome such limitation, chitosan-based nanoparticles are prepared, which improves the stability, absorption rate, intestinal epithelial cell targeting, and prevents TE and TF from oxidative degradation by nanoencapsulation [20,27]. Black tea leaf extract was used as a capping agent to stabilize the silver nanoparticles synthesized by the electrolyte deposition green nanotechnology method. To assess their anti-cancer action against HeLa cervical tumor cell lines, dose-dependent MTT assay was performed, which provided up to 75% of the rate of growth inhibition. Another study involved the synthesis of stable gold and silver nanoparticles with tea extract having TE and TF as a strong antimicrobial activity. Gelatin-based nanoparticles of approximately 200 nm in size, having soft-gel consistency encapsulated with polyelectrolytes, were synthesized using the layer-by-layer technique [56]. Polyphenols released through gelatinized nanoparticles have a strong inhibitory effect on hepatocyte growth factor-induced breast cancer cells. Gold nanoparticles of tea polyphenols synthesized by a green nanotechnological process showed significant action towards prostate and breast cancer cell lines. The two polyphenols, TE and TF, are bulky and stearic compounds present in tea. They play a stabilizing role and act as capping agents in the synthesis of many nanoparticles [57]. Clinical studies of the theaflavins and thearubigins proved these molecules as effective anti-inflammatory, antioxidant, anticancer, and antiosteoporotic agents. In many of the clinical studies, theaflavins and thearubigins rich green tea extracts significantly lowered blood cholesterol levels [75].

An eco-friendly green synthesis nanotechnological approach was reported to improve the solubility, bioavailability, permeability, and stability of polyphenols, and the drugs are outlined in Table 1.

### 3.9. Nano-Quercetin

The bioactive polyphenol quercetin (Qc) has been extensively used for many pharmacological applications, such as anti-inflammatory, anti-Alzheimer, anti-arthritic, wound healing, anti-ischemic, antihypertensive, anti-diabetic, and antioxidant. Qc has also been extensively investigated for anticancer and anti-psoriatic activities. To improve various physicochemical and biopharmaceutical properties of quercetin, a nanotechnological formulation has shown great potential in the pharmaceutical field (see Figure 2 for illustration). The nano-Qc was employed in chemotherapeutic amelioration of apoptosis in cancer cell lines in combination with doxorubicin. The nano-Qc was investigated for antiproliferative effects by MTT assay, and the gene targeting potential was revealed with RT-PCR [22,75]. This combined treatment has been shown to elevate the chemotherapeutic effectiveness of a chemical agent (doxorubicin) by increasing its permeability. A nano-Qc was synthesized with several characterization tests utilized in quorum quenching of *Streptococcus* mutants with the aid of photodynamic therapeutic application. This strategy showed a maximum ROS generation and abolished microbial biofilm and the down regulation of quorum-sensing system genes. Triphenylphosphonium coated nano-Qc was used for ROS-mediated treatment of cerebral ischemia by enhancing cell membrane permeation and improving biopharmaceutical properties, following which the hindrance in mitochondrial targeted delivery was achieved. The oral administration of nano-Qc capsules portrayed a remarkable Qc uptake increase in the brain and controlled mitochondrial delivery, which resulted in amelioration of histopathological severity. Nano-Qc was investigated in arresting mitochondrial damage in ethanolic-induced gastric ulcer rat models. In comparison to Qc, the N-Qc provides higher (up to 20 folds) efficacy in blocking matrix metalloproteinase (MMP)-9 and oxidative stress-generated gastric ulcers [75]. This favorable impact is achieved by increasing Qc bioavailability, which is supplemented by the well-selected nanoformulation components, such as the selection of the polymer, solvent system, surfactant ratios, and method of preparation, resulting in significant biopharmaceutical improvements [76]. A clinical study of nano-quercetin hydrogel on skin wounds of 56 diabetic patients significantly lowered the wound healing time compared to standard drug treatment [77,78]. Some other promising clinical trials regarding human applicability of nano-quercetin have also shown promising pharmacological activities [79,80].

## 4. Nanodeliveries of Dietary Polyphenols and Formulation Considerations

**Figure 3:**
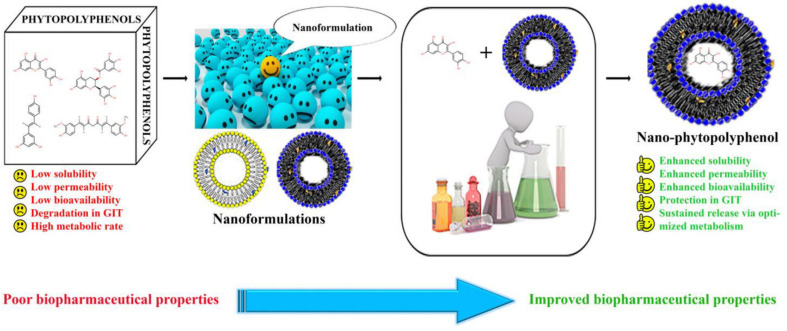
Schematic representation of biopharmaceutical improvements of dietary polyphenols for nanodeliveries.

A schematic representation of biopharmaceutical improvements of phytopolyphenols for effective nanodeliveries is given in Figure 3. An excessive hepatic and systemic metabolism of resveratrol limits the therapeutic applicability of resveratrol. Resveratrol has a poor GIT absorption profile because of low aqueous solubility, consequently, a major area of research is towards modifications in the structure of resveratrol to produce its derivatives, which may impart higher bioavailability when orally taken. One such example is the micronized form of trans-resveratrol (SRT501) liquid formulation, which produced five times higher bioavailability than that of free resveratrol. However, this micronized formulation was not well tolerated by patients and showed some side effects, such as dehydration, diarrhea, vomiting, and also led to renal failure in some cases [81,82,83,84].

Examples of formulation paradigms designed to enhance physicochemical properties of polyphenols in nanodeliveries are given in Table 2.

The most important features of nanodrug delivery systems are orally administered nano-medicines with their capability to overcome the chemical and physical barriers in the gut, such as the acidic pH of the stomach, intestinal mucosal lining, and selectively permeable membranes of enterocytes.

Physico-chemical properties of nanoparticles, such as their metabolism, absorption, distribution, and excretion, depend on their particle size, hydrophobicity, and charge. Particle size is an important parameter of nanomedicines that determines its pharmacokinetics, cell entry, and interaction with the immune system [4].

Surface properties of nanoparticles determine their hydrophilicity or hydrophobicity, and also various biological effects, including cellular uptake, interactions with plasma proteins, particle removal, as well as immune responses. Surface charge is also an important characteristic of nanoparticles which determines cellular absorption and cytotoxicity. Due to their properties, nanoparticles may protect loaded bioactive components from degradation throughout GI digestion and cellular metabolism [5].

Nano-carried bioactive compounds are ultimately released in the GIT, in the circulatory system, and finally, in various tissues. Their subsequent biological facts depend on their chemical and physical properties, as well as the site of release. The location of bioactive release can be driven with nano-materials to allow the release of such therapeutics in particular tissues and specific body sites. The nanostructures can act by either active or passive therapeutic targeting [7,8].

Following the passive nanodrug delivery mode, the loaded therapeutic component is released by the erosion or diffusion of the delivering nano-vector. The active drug delivery mode allows the controlled release of transported biomolecules at the targeted body sites. In this drug delivery mode, certain RNAs, proteins, lipids, carbohydrates, and small metabolite components are used as biomarkers to reach particular target sites. Selective targeting of specific tissues or body sites also becomes possible in this therapeutic modality by incorporating specific stimuli-responsive compounds, which can be triggered by a particular stimulus such as an electric or magnetic field, light, pH, heating, ultrasound, or by contact with concentrated ionic solutions or many enzymes [8,9].

Moreover, there exists a possibility to modify the physical surface properties of metallic nanoparticles, such as gold, silver, and iron oxide nanoparticles, so that they act as drug carriers by the active drug delivery mode. The use of organic nano-carriers is, however, considered to be more preferred because their physicochemical properties can be more finely tuned by modifying their chemical composition, shape, particle size, structural morphology, and surface properties [4,7,8].

The efficiency of nanodrug delivery of natural therapeutic components is also dependent on their molecular weight. However, an increase in the molecular weight generally results in a decrease in the efficiency of the delivery of loaded compounds, resulting in their lower bioavailability [9].

**Figure 4:**
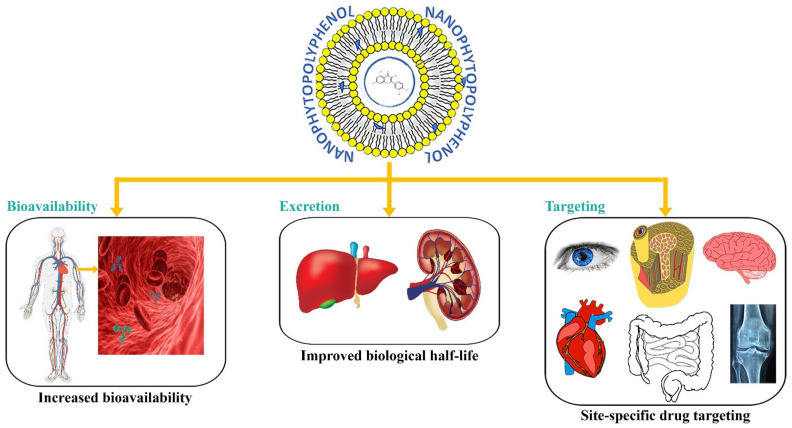
Nanodeliveries of dietary polyphenols and their modified pharmacokinetic profile (bioavailability, metabolism, and targeting).

Different therapeutic nanodrug delivery systems can provide various health benefits depending on their properties, the features of loaded compound/agents, and their desired therapeutic applications. In particular, natural compound-loaded nanoparticles provide many benefits over conventional formulations in terms of therapeutic potential. These benefits include improved epithelium permeability, enhanced stability, extended half-life, increased solubility, bioavailability, and improved tissue targeting, as well as minimized side effects (Figure 4). The nanotechnology-based systems have been increasingly applied to prevent or cure aging-associated (neurodegenerative disorders) pathological conditions (such as Alzheimer’s and Parkinson’s diseases), cancer, cardiovascular diseases, obesity, and type 2 diabetes [3,4,6].

## 5. Patents on Nanoformulations of Phytopolyphenols

Although there are several patents for phytopolyphenol, either solely or in combination with a synthetic drug candidate, only a few studies completed in a research laboratory will reach the global market due to poor physicochemical and biopharmaceutical properties. Table 3 represents the lists of uncommon patents on devices and techniques that have been published or registered, and may represent the future of drug administration via nanocarriers for the discussed diseased conditions [81,82,83,84,85,86,87,88,89].

## 6. Conclusions

Medicines from herbal sources, spices, and dietary supplements have great potential to enhance biological activity and overcome limitations when conjugated with nanotechnology-based drug delivery science. Further, considerable obstacles to the application of clinical therapy in this sector persist. Some of the main problems in transferring these technologies to the industrial world include trials of novel drug delivery strategies to manage the interactions of nanomaterials with biological systems. The development of nanotechnology-based drug delivery systems faces evolving difficulties: the possibility of obtaining multifunctional systems (pilot plant setups) that can accomplish multiple biological and therapeutic activities, as well as the potential of scale-up strategies that bring novel treatment approaches swiftly to the market. Other emerging problems include investigating the targeting efficiency of nanoparticulate drug delivery and meeting international criteria, as well as regulatory aspects for toxicity profiles and biocompatibility.

## Figures and Tables

**Table 1:** Nanoformulation approaches designed for the delivery of dietary polyphenols.

Polyphenol	Type of Nanoformulation	Method of Preparation	Effect(s)
Epigallocatechin-3-gallate (EGCG)	Hydrogels	Protein conjugation	Strong antibacterial activity
	Nanoparticles	Self-assembly of complexes	Active against metastatic pulmonary melanoma
	Chitosan nanoparticles	Gelation method	Improved anti-inflammatory effects
Resveratrol	Liposomes	Thin-film hydration method	Photostability
	Polymeric nanoparticles	Nano-precipitation method	Improved cytotoxicity
	Solid lipid nanoparticles	Hot melt homogenization technique	Increased antioxidant activity
	Cyclodextrins	Freeze drying method	Increased aqueous solubility
Curcumin	Liposomes	Thin-film hydration method	Improved systemic bioavailability
	Polymeric micelles	Solid dispersion method	Inhibitory property against tumor growth
	Lipid-core nanoparticles	Solvent evaporation method	Improved anti-glioma activity
Genistein	Solid lipid nanoparticles	Hot homogenization method	Enhanced bioavailability
	Nanoparticles	Nano-precipitation	Enhanced water solubility and bioavailability
	Layered nano-emulsion	Homogenization and ultrasonication	Extended and targeted release
	Topical nano-emulsion	Spontaneous emulsification	Improved permeation
Kaempferol	Nano-suspension	High-pressure homogenization	Improved bioavailability
	Nanoparticles	Solvent evaporation method	Improved antibacterial activity
Naringenin	Polymeric nanoparticles	Emulsification-diffusion-evaporation	Enhanced bioavailability and cytotoxic effect
	Nano-emulsion	Aqueous phase titration	Controlled release and improved skin regeneration
	Nano-complexes	Thin-film hydration	Improved ocular bioavailability
	Liposomes	Thin-film rehydration	Improved drug loading and oral absorption
Apigenin	Liposomes	Rotary vacuum evaporation method	Improved solubility, bioavailability, and antioxidant activity
	Nano-crystals	Supercritical antisolvent process	Enhanced dissolution and bioavailability
	Carbon nanotubes	Solid dispersion	Improved drug safety and bioavailability
	Polymeric nanoparticles	Solvent evaporation method	Targeted and prolonged drug release

**Table 2:** Formulation paradigms designed to enhance physicochemical properties of polyphenols in nanodeliveries.

Formulation Type	Technique of Preparation	Effect(s)
EGCG -Nanoliposome	Surface coating technology	Above 90% encapsulation efficiency; 100–200 nm in size; more than 70% release
Chitosan-Tea-Catechins NPs	Self-assembly method	More than 72% encapsulation efficiency; 120–150 nm in size; 60% of sustained release in 10 hrs
Chitosan-EGCG Nanoclusters	Ionic interaction method	More than 83% encapsulation efficiency; 300 nm in size
Beta-Chitosan TF-TR NPs	Ionic gelation interaction	More than 97% encapsulation efficiency; less than 90 nm in size; above 80% sustained release in 5 hrs
Pomegranate ellagitannins-NPs	Ionic interaction method	Entrapment efficiency 94%; spherical shape with approximately149 nm in size

**Table 3:** Patents of dietary polyphenols in nano-sized drug delivery systems.

Sl. No.	Patent Number	Formulation Patented	Approach
1	CN109517309A	Nano-cellulose antibacterial self-healing hydrogel	The plant polyphenol nano-cellulose self-healing aquagel system is built using the dynamic covalent bond effect and has strong anti-microbial properties.
2	WO2020088702A1	Curcumin and camptothecin nanocrystals	Nanocrystal production technique is based on ionic compounds creating self-assembled nanostructures and compositions
3	EP3510995A1	Curcumin, resveratrol, vitamin E loaded nanoemulsion	The formulation of different oil-in-water (o/w) nanoemulsion comprising an oil phase or oil core, generally vitamin E or oleic acid, stabilised by a sphingolipid of the sphingomyelin type
4	WO2019227169A1	Core-shell polymer NPs of curcumin, quercetin, silybin, scutellarin and honokiol	This innovation pertains to a method to create a core-shell polymer nanoparticle, the core-shell polymer nanoparticle created as a result of this approach, and its application in the administration of an active ingredient
5	WO2020249383A1	Curcumin, hesperidin, hesperetin, apigenin, epigallocatechin, arctigenin, or glabridin NPs	A technique for creating a delivery system and an active substance delivery system, especially for use in anticancer drug delivery system
6	US11129797B1	Catechin and gingerol metal NPs with surface ligands	A combination of (+)-catechin (2R,3S) as well as gingerol bonded to the exterior of noble metal nanoparticles, with non-oxidative binding via non-deprotonated alcohol and ether functional groups.
7	WO2020122816A1	NPs of hyaluronic acid and EGCG	A dimeric EGCG and hyaluronic acid compound with numerous conjugation sites in its polymer backbone reaches to myeloid leukaemia tumor cells
8	US20210299082A1	Fisetin NPs	Fisetin formulation with increased solubility, primarily to improve its efficacy, potency, and/or bioavailability in treatments
9	US20200197354A1	Quercetin nanomicelle	A formulation containing a freeze-dried quercetin composition in an enclosed environment primarily composed of an inert carrier gas or a mixture of inert gases, the quercetin component containing a drug delivery formulation
10	WO2021015631A1	Polyphenol composed nanofibers	A matrix composed of versatile nanofibres stacked together with at least one or more functional bioactives in each layer to work independently or synergistically for the control of the whole wound cycle

## Data Availability

Not applicable.
